# SIRT5 Alleviates Apoptosis of Vascular Endothelial Cells Under Simulated Microgravity via Desuccinylation of ERO1A

**DOI:** 10.3390/ijms26072908

**Published:** 2025-03-23

**Authors:** Yikai Pan, Qian Zhang, Chengfei Li, Xi Li, Shuhan Li, Yuan Wang, Ruonan Wang, Jieyi Fan, Yateng Tie, Xingcheng Zhao, Yuan Gao, Yongchun Wang, Xiqing Sun

**Affiliations:** 1 Department of Aerospace Medical Training, School of Aerospace Medicine, Fourth Military Medical University, Xi’an 710032, China; panyk0820@163.com (Y.P.); zhangqian00525@163.com (Q.Z.); shuhan_li@139.com (S.L.); wangcircle22@163.com (Y.W.); xiaopinannan@126.com (R.W.); yatengt@163.com (Y.T.); gaoyuan1@fmmu.edu.cn (Y.G.); 2 College of Life Sciences, Yan’an University, Yan’an 716000, China; 3 Key Lab of Aerospace Medicine, Chinese Ministry of Education, Xi’an 710032, China; licf920305@163.com (C.L.); lix1906@163.com (X.L.); yiran9911@163.com (J.F.); zhaoxc@fmmu.edu.cn (X.Z.)

**Keywords:** SIRT5, apoptosis, vascular endothelial cells, microgravity, ERO1A, succinylation

## Abstract

The adverse effects of weightlessness on the human cardiovascular system greatly hinder the process of long-term and long-distance space exploration. Succinylation is an important type of protein post-translational modification. However, whether succinylation modification is able to play a role in altered vascular endothelial cell function under microgravity or simulated microgravity has not been reported. This study aims to investigate the quantitative global proteome and the changes in lysine succinylation in related proteins, seeking to facilitate a better understanding of the protein post-translational modification in cardiovascular deconditioning under microgravity. LC-MS/MS combined with bioinformatics analysis were used to quantitatively detect the perspectives at the global protein level. Immunoprecipitation and Western blot analysis were conducted to further verify the alterations of related proteins and lysine succinylation. A total of 132 differentially expressed proteins and 164 differentially expressed lysine succinylation sites were identified in human umbilical vein endothelial cells (HUVECs). Bioinformatics analysis indicates that lysine succinylation may play a potential role in energy metabolism. In addition, desuccinylase SIRT5 was downregulated and regulated succinylation modification levels of HUVECs under simulated microgravity. Notably, the overexpression of SIRT5 effectively protected HUVECs from apoptosis induced by simulated microgravity. And the succinylation of Lys396 in ERO1A was significantly increased in HUVECs under simulated microgravity. Mechanistically, the knockdown of SIRT5 was found to induce the apoptosis of HUVECs through the succinylation of Lys396 in ERO1A. These results can provide new ideas for elucidating the molecular mechanism of cardiovascular dysfunction in microgravity environments, and provide key molecular targets for scientific protective measures against microgravity in space.

## 1. Introduction

The stress and adaptive changes caused by weightlessness will not only affect the work efficiency of astronauts but also pose a serious threat to their health. Studies have shown that cardiovascular dysfunction caused by weightlessness is the fundamental cause of reduced orthostatic tolerance and decreased exercise capacity in astronauts [1,2]. Revealing the relevant molecular mechanism and putting forward more effective protective measures have become continuing concerns and urgent problems for all aerospace powers. Recent studies have found that abnormal vascular structure and function play an important role in cardiovascular dysfunction caused by weightlessness [3,4], and vascular endothelial cells are a key link in the regulation of vascular function. Under the conditions of weightlessness in space or simulated weightlessness on the ground, the morphological structure and function of vascular endothelial cells are significantly changed [5,6,7].

Protein post-translational modification (PTM) is a dynamic and reversible protein post-translational chemical modification, which plays an important role in protein processing and maturation [8]. It can change the physical and chemical properties of proteins and affect their spatial conformation and stability [9]. It is widely present in prokaryotic and eukaryotic cells, participates in various life activities, and plays a key regulatory role. Common PTMs include phosphorylation, methylation, acetylation, ubiquitination, glycosylation, and succinylation. Among them, succinylation modification is relatively conservative in the process of biological evolution and is involved in almost all biological processes of living organisms [10]. Through protein succinylation modification, succinyl donors covalently bind succinyl groups to residues of lysine enzymatically or nonenzymatically to induce charge mutations [11]. Compared with other PTMs, succinylation modification can cause more changes in physicochemical properties and functions of proteins; participate in cellular oxidative stress [12], metabolic regulation [13], and signal transduction [14]; and are closely related to the occurrence and development of a variety of human diseases, which has become a research hotspot in the field of life science.

Studies have shown that the level of succinylation modification is mainly regulated by succinyltransferase, succinyl donors, and desuccinylase [15]. Among them, succinyltransferase and succinyl donors play a positive regulatory role in succinylation modification, while desuccinylase plays a negative regulatory role in succinylation modification [16]. The sirtuin (SIRT) family belongs to nicotinamide adenine dinucleotide (NAD+)-dependent class III deacetylases, including seven members of SIRT1–7. Compared with other members of the SIRT family, SIRT5 only has a very weak deacetylase activity due to its structural characteristics of a larger lysine acyl binding pocket, while it also has a strong desuccinylase activity [17]. Succinylation modification is widespread in the living world, and most of the succinylation modification occurs in mitochondria, which is closely related to mitochondrial energy metabolism [18]. It participates in and regulates multiple metabolic signaling pathways including the tricarboxylic acid cycle, amino acid metabolism, and fatty acid oxidation, and then affects the structure and function of mitochondria [19,20]. The oxidative metabolism of mitochondria can generate large amounts of reactive oxygen species (ROS). Low levels of ROS can act as redox agents, but excessive ROS can damage macromolecules in cells and may lead to apoptosis [21].

Unfortunately, to date, few studies have specifically focused on the alterations of the quantitative global proteome and lysine succinylation in HUVECs under simulated microgravity. We thus undertook this study to investigate the quantitative global proteome and the changes in lysine succinylation in related proteins, seeking to facilitate a better understanding of the molecular mechanisms underlying cardiovascular dysfunction in microgravity environments.

## 2. Results

### 2.1. Increase in Lysine Succinylation in HUVECs Under Simulated Microgravity

**Figure 1:**
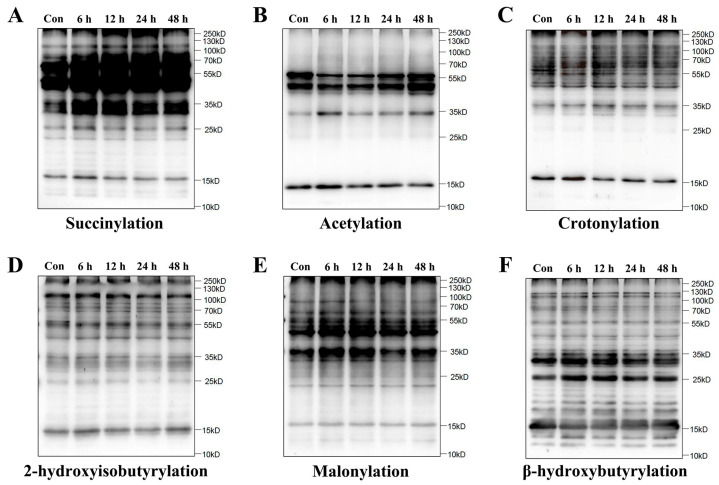
Increase in lysine succinylation in HUVECs under simulated microgravity. A-F. Representative Western blot of lysine succinylation (**A**), acetylation (**B**), crotonylation (**C**), 2-hydroxyisobutyrylation (**D**), malonylation (**E**), and β-hydroxybutyrylation (**F**) in HUVECs.

To determine whether lysine succinylation was present in HUVECs, we examined the global profiles of six PTM types in HUVECs after simulated microgravity by Western blot assay, including succinylation, acetylation, crotonylation, 2-hydroxyisobutyrylation, malonylation, and β-hydroxybutyrylation. As expected, a high abundance of lysine succinylation was observed in microgravity groups relative to the control group (Con group), while other PTMs did not change remarkably (Figure 1).

### 2.2. Identification of Lysine-Succinylated Proteins and Sites in HUVECs Under Simulated Microgravity

**Figure 2:**
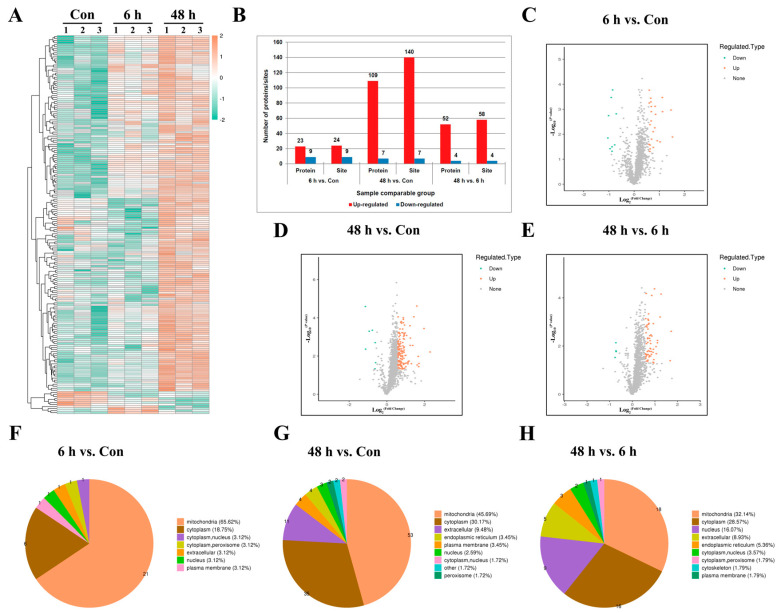
Identification of lysine-succinylated proteins in HUVECs. (**A**) The thermal map shows the differentially succinylated sites in HUVECs under simulated microgravity compared with the Con group (|log2FC| > 0.585, *p* < 0.05). (**B**) Identified protein site number distributions in HUVECs. (**C–E**) Volcano plots of differentially succinylated sites and protein in HUVECs. (**F–H**) Subcellular localizations of lysine-succinylated proteins in HUVECs.

Based on the relative quantitative values of differential modification sites, a differential modification site heatmap (Figure 2A) can be plotted. Compared with the Con group, a significant upregulation of modification levels was identified at 164 sites on 132 proteins. The modification levels of 16 sites on 16 proteins were significantly downregulated. Compared with the simulated weightlessness group for 6 h, the simulated weightlessness group for 48 h showed a significant upregulation of 58 modification sites, distributed in 52 proteins, and a significant downregulation of 4 modification sites, distributed in 4 proteins (Figure 2B). And a differential modification site volcano map (Figure 2C−E) can be further plotted. WoLF PSORT can be used to annotate the subcellular structure of protein expression sites in cells. As a result, it was found that compared with the Con group, the proportion of differentially expressed succinylation modifications of mitochondrial proteins in simulated weightlessness groups was highest (Figure 2F−H).

### 2.3. Functional Enrichment Analysis of Lysine-Succinylated Proteins

**Figure 3:**
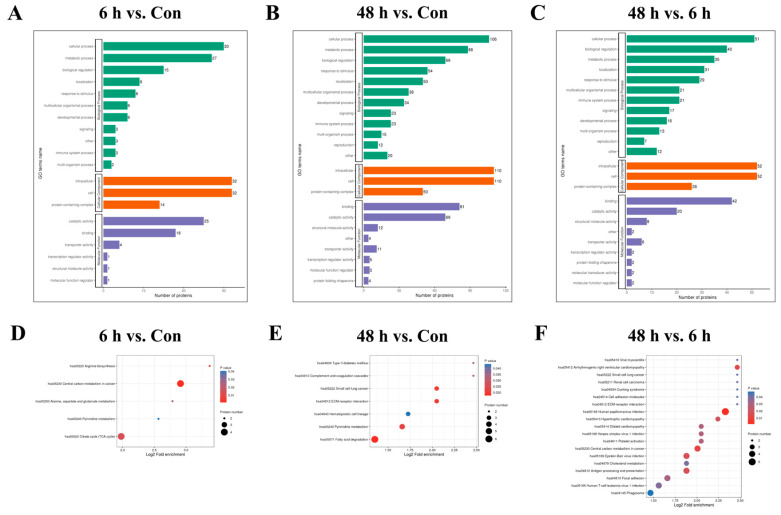
Functional enrichment analysis of lysine-succinylated proteins. GO (**A–C**) and KEGG (**D–F**) enrichment analyses in HUVECs after simulated microgravity compared with those in the Con group.

We then performed GO enrichment analysis on differentially modified proteins in the simulated weightlessness group for 6 h or 48 h to understand the biological processes, cellular components, and molecular functions involved in the corresponding proteins. The results (Figure 3A−C) showed that differentially modified proteins among the Con and 6 h and 48 h simulated microgravity groups may be involved in cellular respiration, inflammation regulation, vesicle transport, cytoskeleton formation, acyltransferase activity, and ion channel activation. Enrichment analysis of the KEGG pathways involved in differentially modified proteins revealed that the main pathways involved include the TCA cycle, cell adhesion, fatty acid and cholesterol metabolism, etc. (Figure 3D−F).

### 2.4. Motif Analysis of Lysine-Succinylated Proteins

**Figure 4:**
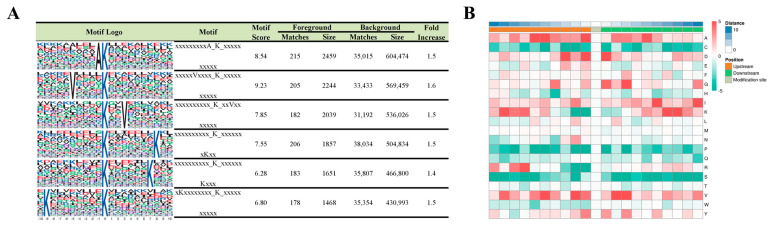
Motif analysis of the detected succinylation sites. (**A**) Succinylation motifs for amino acid sequences surrounding the lysine-succinylated sites (−10 to +10). The letter height represents the frequency of that amino acid residue at that position. The K in the middle position corresponds to the succinylated lysine. (**B**) Heat map analysis of the amino acid compositions around the succinylated sites. Red indicates an amino acid that is significantly enriched, whereas green indicates an amino acid that is significantly reduced.

To examine whether there were specific motifs for lysine succinylation in HUVECs under simulated microgravity, we generated WebLogo sequence motifs and IceLogo heat maps. Motif-X was used to analyze the amino acid sequences from −10 to +10 of all identified succinylation sites. Six conserved motifs surrounding the succinylated lysine (K) residues were identified, including xxxxAK, VxxxxK, xxKxxV, KxxxxxxxK, KxxxxxxK, and KxxxxxxxxK (x indicates a random amino acid residue) (Figure 4A). The heat map assessed the relative frequencies of each residue in the position of a 21 amino-acid-long sequence context (Figure 4B).

### 2.5. SIRT5 Is Downregulated and Regulates Succinylation Modification Levels of HUVECs Under Simulated Microgravity

**Figure 5:**
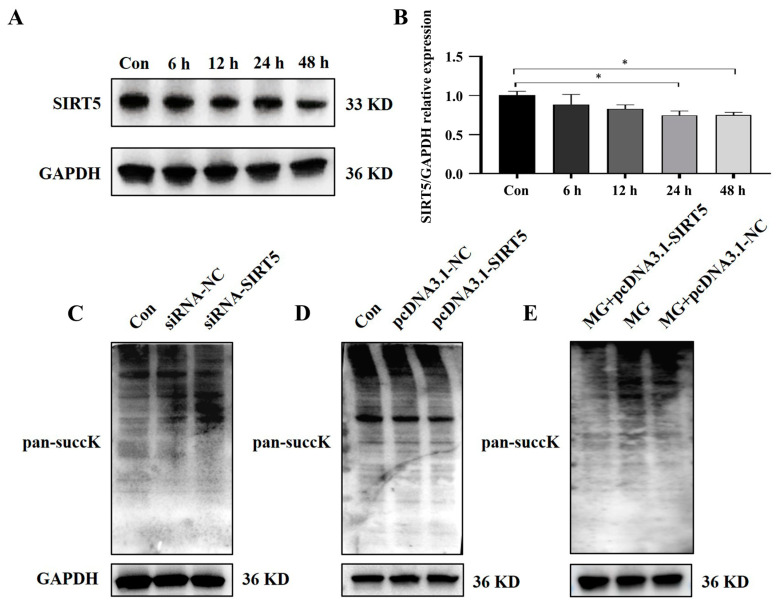
SIRT5 is downregulated and regulates succinylation modification levels of HUVECs under simulated microgravity. (**A**) The representative Western blot of SIRT5 protein expression in HUVECs after simulated microgravity; GAPDH was used as the loading control. (**B**) Statistical analysis of the Western blot. (**C–E**) Representative Western blots of the succinylated protein expression in HUVECs affected by SIRT5; GAPDH was used as the loading control. * *p* < 0.05. Data are expressed as the mean ± SD, *n* = 3 in each group.

After simulating microgravity with the cell models, changes in SIRT5 expression were evaluated by measuring protein levels. Western blot results demonstrated a notable decrease in SIRT5 protein expression in HUVECs after simulated microgravity (Figure 5A,B). These findings collectively indicated that SIRT5 is downregulated in HUVECs induced by weightlessness. Additionally, we found that changes in SIRT5 expression can affect the level of succinylation modification in HUVECs (Figure 5C−E).

### 2.6. SIRT5 Protects HUVECs from Apoptosis Induced by Simulated Microgravity

**Figure 6:**
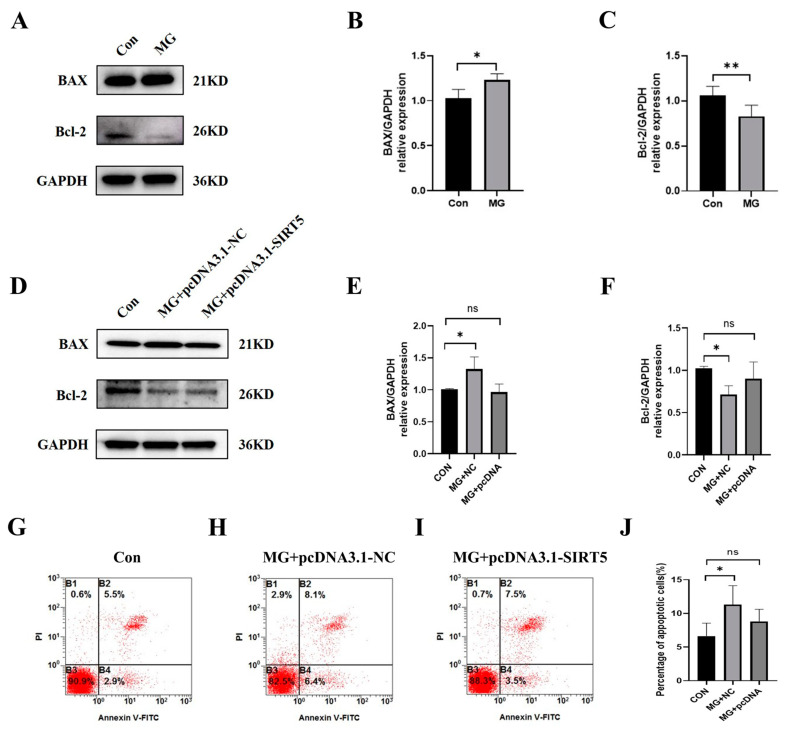
SIRT5 protects HUVECs from apoptosis induced by simulated microgravity. (**A**) Representative Western blots of apoptosis-related protein expression in HUVECs after simulated microgravity; GAPDH was used as the loading control. (**B**,**C**) Statistical analysis of the Western blot. (**D**) Representative Western blots of apoptosis-related protein expression in HUVECs after transfection with pcDNA3.1-SIRT5 or its NC for 48 h; GAPDH was used as the loading control. (**E**,**F**) Statistical analysis of the Western blot. (**G–I**) Representative flow cytometry analysis in HUVECs after transfection with pcDNA3.1-SIRT5 or its NC for 48 h. (**J**) Statistical analysis of the percentage of apoptotic cells. ns, not significant, * *p* < 0.05, ** *p* < 0.01. Data are expressed as the mean ± SD, *n* = 3 in each group.

To evaluate the effects of SIRT5 in the apoptosis of HUVECs, we used pcDNA-NC and pcDNA3.1-SIRT5 to transfect cells. The results of Western blot (Figure 6A−F) and flow cytometry analysis (Figure 6G−J) illustrated that the upregulation of SIRT5 decreased apoptosis in HUVECs under simulated microgravity. These results suggested that promoting SIRT5 expression under simulated microgravity can reduce HUVEC apoptosis.

### 2.7. Simulated Microgravity Increases the Succinylation of ERO1A Protein at Site K396

**Figure 7:**
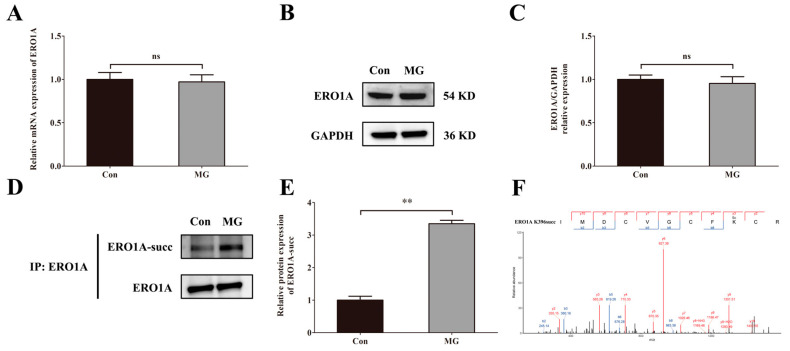
Simulated microgravity increases the succinylation of ERO1A protein at site K396. (**A**) The mRNA expression of ERO1A in HUVECs. (**B**) Representative Western blots of ERO1A protein expression in HUVECs after simulated microgravity; GAPDH was used as the loading control. (**C**) Statistical analysis of the Western blot. (**D**) Representative IP and Western blots of ERO1A-succ in HUVECs after simulated microgravity; ERO1A was used as the loading control. (**E**) Statistical analysis of the IP and Western blots. (**F**) The mass spectrum of succinylated Lys396 of ERO1A in the succinylomics of HUVECs. ns, not significant, ** *p* < 0.01. Data are expressed as the mean ± SD, *n* = 3 in each group.

We measured the mRNA level, protein level of ERO1A, and succinylated ERO1A in HUVECs after simulated microgravity. The results showed that there were no significant changes in the mRNA level (Figure 7A) and protein level of ERO1A (Figure 7B,C) under simulated microgravity environments. However, the level of succinylation modification of ERO1A protein was increased significantly when induced by simulated microgravity (Figure 7D,E). We then predicted the potential site of ERO1A succinylation and identified the K396 site (Figure 7F).

### 2.8. Knockdown of SIRT5 Induces Apoptosis of HUVECs Through the Succinylation of Lys396 in ERO1A

**Figure 8:**
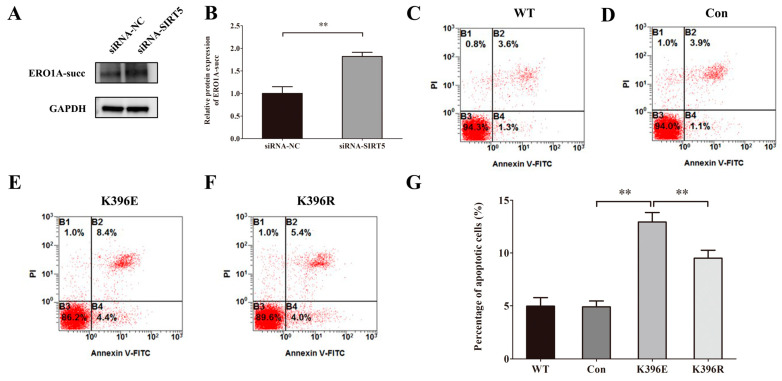
Knockdown of SIRT5 induces apoptosis of HUVECs through the succinylation of Lys396 in ERO1A. (**A**) Representative Western blots of ERO1A-succ protein expression in HUVECs after simulated microgravity; GAPDH was used as the loading control. (**B**) Statistical analysis of the Western blot. (**C–F**) Representative flow cytometry analysis in HUVECs after transfection with WT, Con, K396E, or K396R for 48 h. (**G**) Statistical analysis of the percentage of apoptotic cells. ** *p* < 0.01. Data are expressed as the mean ± SD, *n* = 3 in each group.

To analyze the correlation between SIRT5 and its downstream target, ERO1A, we used siRNA-NC and siRNA-SIRT5 to transfect cells. The results of Western blot showed that the downregulation of SIRT5 increased the protein level of ERO1A-succ significantly (Figure 8A,B). To investigate the effect of Lys396 succinylation on the apoptosis of HUVECs, we used plasmids of WT, Con, K-to-E mutant, and K-to-R mutant to transfect cells. The results of flow cytometry analysis (Figure 8C−G) illustrated that K396E increased HUVEC apoptosis and K396R decreased HUVEC apoptosis, compared with Con or K396E groups.

## 3. Discussion

The major hypothesis of this study is that ground-based simulated microgravity would alter succinylation expression profiles in HUVECs and SIRT5 would take part in the apoptosis of HUVECs. We used 2D-clinostat and proteome technology to study the effect of simulated microgravity on HUVECs. The results indicate that some protein sites of succinylation modification seem to be repressed or activated under experimental conditions. In addition, we demonstrated the role of SIRT5 in vascular endothelial cells apoptosis. This may present a new explanation to the changes of morphology and function in HUVECs under simulated microgravity.

SIRT5 is a desuccinylase that functions by catalyzing the removal of succinylation, thereby inhibiting protein succinylation. Evidence has revealed the potential roles of SIRT5 in various human heart diseases. Importantly, SIRT5’s impact on I/R-induced myocardial injury has been previously documented [22]. However, whether SIRT5 affects apoptosis in HUVECs under simulated microgravity through the desuccinylation of its target proteins remains unclear. In this study, we confirmed that SIRT5 was significantly downregulated in the weightless cell model. To analyze SIRT5’s involvement in apoptosis in HUVECs, we overexpressed SIRT5 and conducted various detections. Our experimental results indicated that SIRT5 overexpression protected cells from apoptosis. These findings confirm the role of SIRT5 in alleviating the apoptosis of HUVECs under simulated microgravity.

Mechanistically, SIRT5 regulates protein desuccinylation to exert its functions across different disease models. For instance, SIRT5 mediates the desuccinylation of ACOX1, decreasing its activity in hepatocellular carcinoma development [23]. In the context of intervertebral disc degeneration, SIRT5 protects mitochondrial homeostasis via the desuccinylation modification of AIFM1 [24]. Additionally, SIRT5 enhances cellular antioxidant defense by promoting the desuccinylation of IDH2 and eliminates reactive oxygen species (ROS) through the desuccinylation-mediated activation of SOD1 [25]. However, the interaction between SIRT5 and ERO1A had not been revealed until now. This study demonstrates that SIRT5 overexpression reduces the level of succinylated ERO1A, indicating a regulatory interaction between SIRT5 and ERO1A.

As a member of the sirtuin family of proteins, SIRT5 is an NAD+-dependent desuccinylase that plays a crucial role in cellular metabolism and protein homeostasis. It has been shown to modulate the levels of ERO1A, a key enzyme involved in the oxidative folding of proteins in the endoplasmic reticulum [26], through its desuccinylation activity. Under conditions of cellular stress or hypoxia, ERO1A expression is upregulated to cope with the increased demand for protein folding and disulfide bond formation [27,28]. SIRT5, by removing succinyl groups from ERO1A, can potentially regulate its activity and stability, thereby influencing the cellular response to hypoxic conditions. This desuccinylation event is significant as it implies that SIRT5 may act as a rheostat, controlling the levels and activity of ERO1A, and thus impacting the unfolded protein response and cellular redox homeostasis. The precise mechanism by which SIRT5 regulates ERO1A is an area of active research, with implications for understanding the cellular adaptation to hypoxic stress and the development of therapeutic strategies targeting hypoxia-driven diseases.

## 4. Materials and Methods

### 4.1. Cell Culture and Weightless Model Establishment

HUVECs were purchased from American Type Culture Collection (ATCC, Manassas, VA, USA) and cultured in DMEM (Gibco, Grand Island, NY, USA) containing 10% fetal bovine serum (FBS, Gibco, Grand Island, NY, USA). The cells were maintained at 37 °C in a humidified incubator of 5% CO_2_. The method to establish a weightless cell model was described previously [29]. In short, the cells were seeded and cultured on the coverslips, which were fixed in the chambers filled with complete DMEM. Then, the chambers were placed in the 2D-clinostat (developed by China Astronaut Research and Training Center, Beijing, China) rotating at 30 rpm to simulate microgravity.

### 4.2. Cell Transfection and Treatment

Small interfering RNA (siRNA) targeting SIRT5 (siRNA-SIRT5), SIRT5 overexpression vector (pcDNA3.1-SIRT5), and their corresponding negative controls (siRNA-NC or pcDNA3.1-NC) were all synthesized by GenePharma Biotechnology, Shanghai, China. All plasmids and siRNAs were transfected into HUVECs using lipofectamine 2000 (Invitrogen, Carlsbad, CA, USA) according to the manufacturer’s protocol and consensus guidelines. The transfection efficiencies of overexpression or knockdown were evaluated by qRT-PCR or Western blot.

### 4.3. Protein Extraction and Tryptic Digestion

HUVECs were first ground using liquid nitrogen. Subsequently, they were denatured in a lysis buffer comprising 8 M urea, 1% protease inhibitor cocktail, 3 μM trichostatin A (TSA), and 50 mM nicotinamide (NAM). The mixture was then sonicated three times on ice. After centrifugation at 12,000× *g* for 10 min at 4 °C, the supernatant was collected, and the protein concentration was measured using the BCA assay. For tryptic digestion, the protein solution was precipitated with 20% trichloroacetic acid at 4 °C for 2 h. The precipitates were washed three times with ice-cold acetone following centrifugation at 4500× *g* for 5 min. The dried protein samples were then diluted in 200 mM tetraethylammonium bromide and digested overnight with a trypsin-to-protein ratio of 1:50. Each sample was subsequently reduced with 5 mM dithiothreitol for 30 min at 56 °C and alkylated with 11 mM iodoacetamide for 15 min in the dark.

### 4.4. Lysine-Succinylated Peptide Affinity Enrichment

To enrich succinylated peptides, tryptic peptides were first dissolved in NETN buffer (100 mM NaCl, 1 mM EDTA, 50 mM Tris-HCl, 0.5% NP-40, pH 8.0). The mixture was then incubated with pre-washed anti-succinyl lysine beads (PTM-402, PTM Biolabs, Hangzhou, China) overnight at 4 °C with gentle shaking. After incubation, the beads were washed four times with NETN buffer and four times with deionized water to remove unbound peptides. The specifically bound peptides were subsequently eluted from the beads using 0.1% trifluoroacetic acid. The eluted fractions were combined and dried under a vacuum. Finally, the resulting peptides were desalted using C18 ZipTips for further label-free quantitative analysis.

### 4.5. LC-MS/MS

HUVECs were analyzed simultaneously for both the global proteome and succinylome using 4D label-free quantitation. The tryptic peptides were dissolved in solvent A (0.1% formic acid, 2% acetonitrile). The separation was carried out using a homemade reversed-phase analytical column (25 cm long, 75/100 μm inner diameter) in the NanoElute UHPLC system (Bruker Daltonics, Karlsruhe, Germany) at a constant flow rate of 450 nL/min. For the global proteome analysis, the liquid phase gradient was set as follows: 6–24% solvent B (0.1% formic acid in acetonitrile) for 70 min, 24–35% solvent B for 14 min, 35–80% solvent B for 3 min, and 80% solvent B for 3 min. For the succinylome analysis, the gradient was adjusted to 7–24% solvent B for 40 min, 24–32% solvent B for 12 min, 32–80% solvent B for 4 min, and 80% solvent B for 4 min. Mass spectrometric analysis was performed using a timsTOF Pro mass spectrometer (Bruker Daltonics, Karlsruhe, Germany) in parallel accumulation serial fragmentation (PASEF) mode. The capillary source was operated at an electrospray voltage of 1.65 kV. Precursors with charge states ranging from 0 to 5 were selected for fragmentation, with a dynamic exclusion time set to 24 s. The LC-MS/MS data were acquired with 10 PASEF MS/MS scans per cycle, and the MS spectra were recorded over an *m/z* range of 100–1700.

### 4.6. Database Search

The MS/MS spectral data obtained were analyzed using the MaxQuant search engine (version 1.6.15.0). The tandem mass spectra were searched against the human SwissProt database (containing 20,422 entries) combined with a reverse decoy database. Trypsin/P was specified as the cleavage enzyme, allowing up to 2 missed cleavages. The mass tolerance was set at 20 ppm for the initial search and 5 ppm for the main search of precursor ions, while the tolerance for fragment ions was set at 0.02 Da. Carbamidomethylation on cysteine residues was defined as a fixed modification, whereas oxidation on methionine residues and N-terminal acetylation were set as variable modifications. The false discovery rate (FDR) was controlled to be less than 1%.

### 4.7. Bioinformatic Analysis

The analysis of the sequence model for succinylation sites was conducted using Motif-X software (version 1.2). Specifically, the sequences of 21 amino acids (including 10 residues upstream and 10 residues downstream of the lysine succinylation site) were analyzed, with all protein sequences used as the background and default parameters applied. The amino acid composition surrounding the succinylation sites was visualized using an IceLogo heatmap generated by IceLogo software (version 1.0.2). Subcellular localization predictions were made using WoLF PSORT (https://wolfpsort.hgc.jp/, accessed on 24 June 2024). Gene Ontology (GO) annotations for different categories (cellular component, biological process, and molecular function) were retrieved from the UniProt-GOA database (https://www.ebi.ac.uk/GOA/, accessed on 26 June 2024). For proteins that could not be annotated by UniProt-GOA, GO functions were annotated using InterProScan software (version 5.73-104.0) (https://www.ebi.ac.uk/interpro/, accessed on 24 July 2024). Kyoto Encyclopedia of Genes and Genomes (KEGG) analysis was performed to identify enriched pathways using a two-tailed Fisher’s exact test. Protein–protein interaction (PPI) networks for the identified succinylated proteins were constructed using the STRING database (https://string-db.org/, accessed on 12 August 2024) and visualized using Cytoscape software (version 3.6.1).

### 4.8. Western Blot Assay

The expression levels of apoptosis-related proteins, SIRT5 and ERO1A, were determined by Western blot. Total protein was extracted from HUVECs with cell lysis buffer containing phenylmethylsulphonyl fluoride (PMSF, 1 mM), followed by centrifugation at 10,000× *g* at 4 °C for 15 min. The concentration of protein was determined by using the Pierce BCA Protein Assay Kit (Thermo Fisher, Waltham, MA, USA). Then, the protein samples were mixed with loading buffer, boiled, electrophoresed by 12% sodium dodecyl sulfate polyacrylamide gel electrophoresis gels, and transferred to the polyvinylidene difluoride membranes using the semi-dry transfer method. The membranes were blocked using 5% non-fat milk in a Tris-buffered saline-Tween 20 (TBST) solution at room temperature for 1.5 h. The primary antibodies, including SIRT5 (1:1000), Bax (1:2000), Bcl-2 (1:2000), ERO1A (1:2000), and GAPDH (1:2000) (Cell Signaling Technology, Danvers, MA, USA), were diluted and the membrane was then incubated at 4 °C overnight. The secondary antibody goat anti-rabbit horseradish peroxidase conjugate (Abcam, Cambridge, UK) was diluted 1:5000. The membrane was then incubated with the secondary antibodies at room temperature for 1 h. The protein was visualized by an ECL (enhanced chemiluminescence) detection kit (Amersham Biosciences, London, UK).

### 4.9. Coimmunoprecipitation (Co-IP)

Co-IP experiments were performed to verify the binding relationship between proteins of interest in HUVECs. The Co-IP complexes were purified by the immunoprecipitation kit with protein A + G magnetic beads (Beyotime Biotechnology, Shanghai, China). The dosage of the IP antibody was 5 μg of anti-ERO1A (Proteintech, Wuhan, China). The proteins were harvested and analyzed by Western blot assays.

### 4.10. Apoptosis Assay

Flow cytometry with Annexin V-FITC/PI was performed to detect the apoptosis rates of HUVECs. The treated cells were digested with 0.25% trypsin and washed with cold PBS three times. Then, the cells were resuspended in binding buffer and incubated in a solution mixed with Annexin V-FITC and PI at room temperature (20–25 °C) away from light for 15 min. The apoptosis rates were detected by the FACS Aria II flow cytometer (Becton Dickinson, Franklin Lakes, NJ, USA).

### 4.11. Statistical Analysis

Experimental data obtained from three independently repeated samples are presented as the mean ± standard deviation. Statistical comparisons of the results were analyzed by using Student’s t test for two groups or one-way ANOVA for multiple groups. Differences were considered statistically significant when *p* < 0.05. All statistical analyses were performed with the software SPSS 26.0 (IBM, Armonk, NY, USA).

## 5. Conclusions

In summary, our results first demonstrate that significant changes in succinylation modification levels of endothelial cells have been observed under simulated microgravity conditions, which are associated with the desuccinylation activity of SIRT5. SIRT5, a mitochondrial enzyme known for its desuccinylase function, plays a crucial role in regulating the succinylation status of proteins, including ERO1A. By targeting and removing succinyl groups from ERO1A, SIRT5 modulates the succinylation level of ERO1A, thereby affecting its activity. The altered activity of ERO1A, in turn, influences the occurrence of apoptosis in cells. This regulatory mechanism is significant as it highlights the role of SIRT5 in cellular responses to stress and the maintenance of cellular homeostasis, particularly under conditions that mimic the effects of microgravity on cellular biology.

## Data Availability

The data that support the findings of this study are available from the corresponding author upon reasonable request.
